# Endoscope-assisted microsurgical resection of a primary oculomotor nerve schwannoma involving the cavernous sinus via a pterional approach: a case report with narrative review

**DOI:** 10.3389/fonc.2026.1854683

**Published:** 2026-09-01

**Authors:** Chong Zhang, Zhipeng Wu, Yuyang Zhao, Yujing Zhao, Zhe Wang, Bing Chen

**Affiliations:** 1 School of Clinical Medicine, Shandong Second Medical University, Weifang, Shandong, China; 2 Department of Neurosurgery, Weifang People’s Hospital, Shandong Second Medical University, Weifang, Shandong, China

**Keywords:** case report, cavernous sinus, endoscope-assisted microsurgery, oculomotor nerve schwannoma, pterional approach, stereotactic radiosurgery

## Abstract

Primary oculomotor nerve schwannomas that involving the cavernous sinus represent an exceptionally rare entity. The surgical management of these tumors is complicated by the need to maximize resection while safeguarding oculomotor function. We report a middle-aged female who presented with headache, dizziness, and visual decline. Examination showed right ptosis and decreased visual acuity. MRI demonstrated a lesion in the right parasellar region that involved the cavernous sinus, circumferentially surrounded the cavernous segment of the internal carotid artery, and extended into the optic canal. A right pterional craniotomy was performed with a microsurgical resection under endoscope assistance. Following microsurgical debulking through the oculomotor trigone and supratrochlear triangle, neuroendoscopy was used to explore and curette deep residual tumor within the cavernous sinus. Histopathology confirmed schwannoma of Schwann cell origin. Postoperatively, ptosis showed partial improvement and visual acuity showed mild improvement, with oculomotor and pupillary function preserved without postoperative deterioration. Postoperative MRI obtained within 72 hours confirmed an intentional subtotal resection with a minimal residual enhancing focus, for which adjuvant stereotactic radiosurgery has been recommended. In this patient, endoscopic visualization facilitated inspection of areas not directly visible through the microscope and supported an intentional function-preserving resection. The surgical strategy illustrated in this case may inform the management of this rare pathology, although long-term outcomes remain to be evaluated.

## Introduction

Benign tumors derived from Schwann cells, schwannomas constitute roughly 8% of primary intracranial neoplasms (1). Among these, vestibular schwannomas (acoustic neuromas) are the most common, representing over 90% of intracranial schwannomas (2). An exceedingly rare intracranial lesion, primary oculomotor nerve schwannoma (ONS) has been documented only in sporadic reports across the literature (3). The etiology of ONS remains unclear; most reported cases appear sporadic. An association with neurofibromatosis has been described in a limited number of cases, but the available literature does not establish a consistent link specifically with NF1 or NF2 (4). The clinical presentation of ONS is predominantly characterized by oculomotor nerve palsy, including ptosis, ocular motility deficits, and mydriasis. Some patients may also present with headache. Tumor-related optic nerve compression may result in progressive visual decline (5, 6).

The cavernous sinus is an anatomically complex space that houses the internal carotid artery, the oculomotor nerve (CN III), the trochlear nerve (CN IV), the abducens nerve (CN VI), and branches of the trigeminal nerve (CN V). It has long been regarded as a neurosurgical “no man’s land” (7, 8). Conventional microsurgery is hampered by blind spots, whereas the neuroendoscope permits close-up inspection and panoramic illumination. The microsurgical resection with endoscope assistance holds promise for achieving more complete tumor resection while preserving neurological function (9).

Herein, we report a case of primary oculomotor nerve schwannoma with cavernous sinus involvement. The patient presented preoperatively with headache, dizziness, bilateral visual decline (more pronounced on the right), and right-sided ptosis. We performed a right pterional craniotomy and entered the cavernous sinus through the oculomotor trigone and supratrochlear triangle. Neuroendoscopic assistance facilitated a near-total removal of the tumor. After surgery, the patient’s right ptosis showed partial improvement; vision improved bilaterally, extraocular movements remained full, and pupillary reflexes were intact. Histopathologic examination coupled with immunohistochemical staining verified the lesion to be a schwannoma. Adjuvant stereotactic radiosurgery has been recommended for the residual enhancing focus identified on postoperative imaging. By presenting this case and summarizing the relevant literature, we aim to illustrate the utility of endoscope-assisted microsurgery for ONS with cavernous sinus involvement.

## Case report

A 44-year-old female patient was admitted with a six-month history of headache and dizziness, accompanied by progressive bilateral visual deterioration over the preceding three months.

### Neurological examination

The patient was alert and oriented with fluent speech. Right-sided ptosis was noted, with the vertical palpebral fissure height measuring approximately 5 mm on the right and 7 mm on the left. Bilateral pupils were equal, round, and approximately 3.0 mm in diameter, with brisk direct and consensual light reflexes. Visual acuity was diminished bilaterally: 0.8 in the right eye and 0.9 in the left eye, with more pronounced impairment on the right. Extraocular movements were full in both eyes. Examination of the remaining cranial nerves was unremarkable. Muscle strength was Medical Research Council grade 5/5 in all four extremities, muscle tone was generally normal, and sensory and cerebellar functions were intact. Pathological reflexes were negative.

### Imaging studies

**Figure 1:**
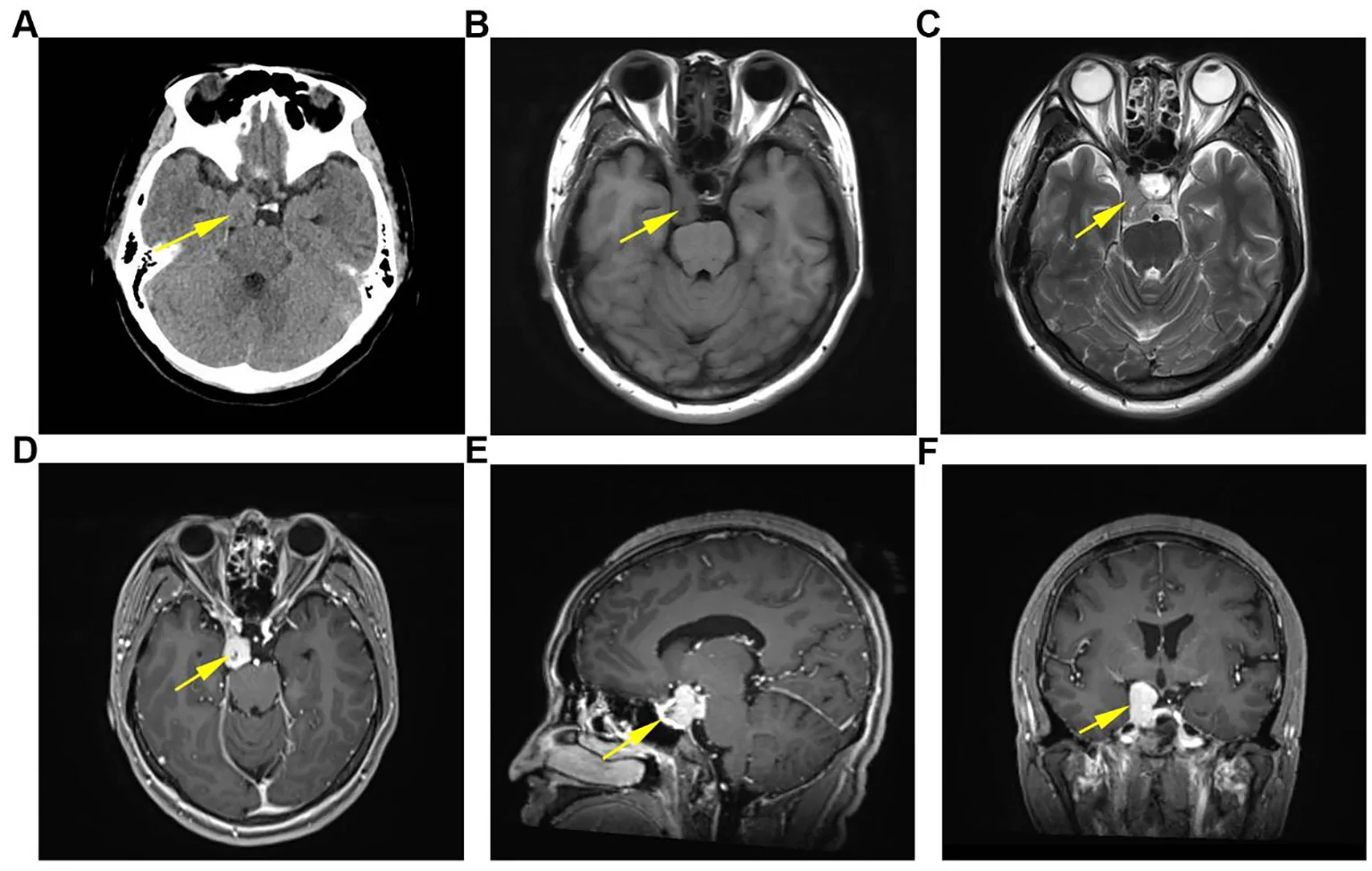
Preoperative imaging. **(A)** Axial non-contrast CT (brain window). **(B)** Axial T1WI. **(C)** Axial T2WI. **(D)** Axial CE-T1WI. **(E)** Sagittal CE-T1WI. **(F)** Coronal CE-T1WI.

Admission non-contrast cranial CT revealed a hypodense lesion with ill-defined borders in the right parasellar region (**Figure 1A**). Brain MRI with gadolinium showed a space-occupying mass in the right sellar area, measuring roughly 3.2 × 1.5 × 2.1 cm. The lesion displayed mixed iso- to hypointensity on T1WI and hyperintensity on T2WI, with avid post-contrast enhancement of the solid portion and scattered non-enhancing cystic foci. Tumor extension was evident anteriorly into the right optic canal and posteriorly toward the right pontine surface, lying adjacent to the brainstem. The mass also circumferentially encased the cavernous segment of the right internal carotid artery (C4 segment according to the Bouthillier classification), causing focal stenosis (**Figures 1B–F**).

### Treatment course

**Figure 2:**
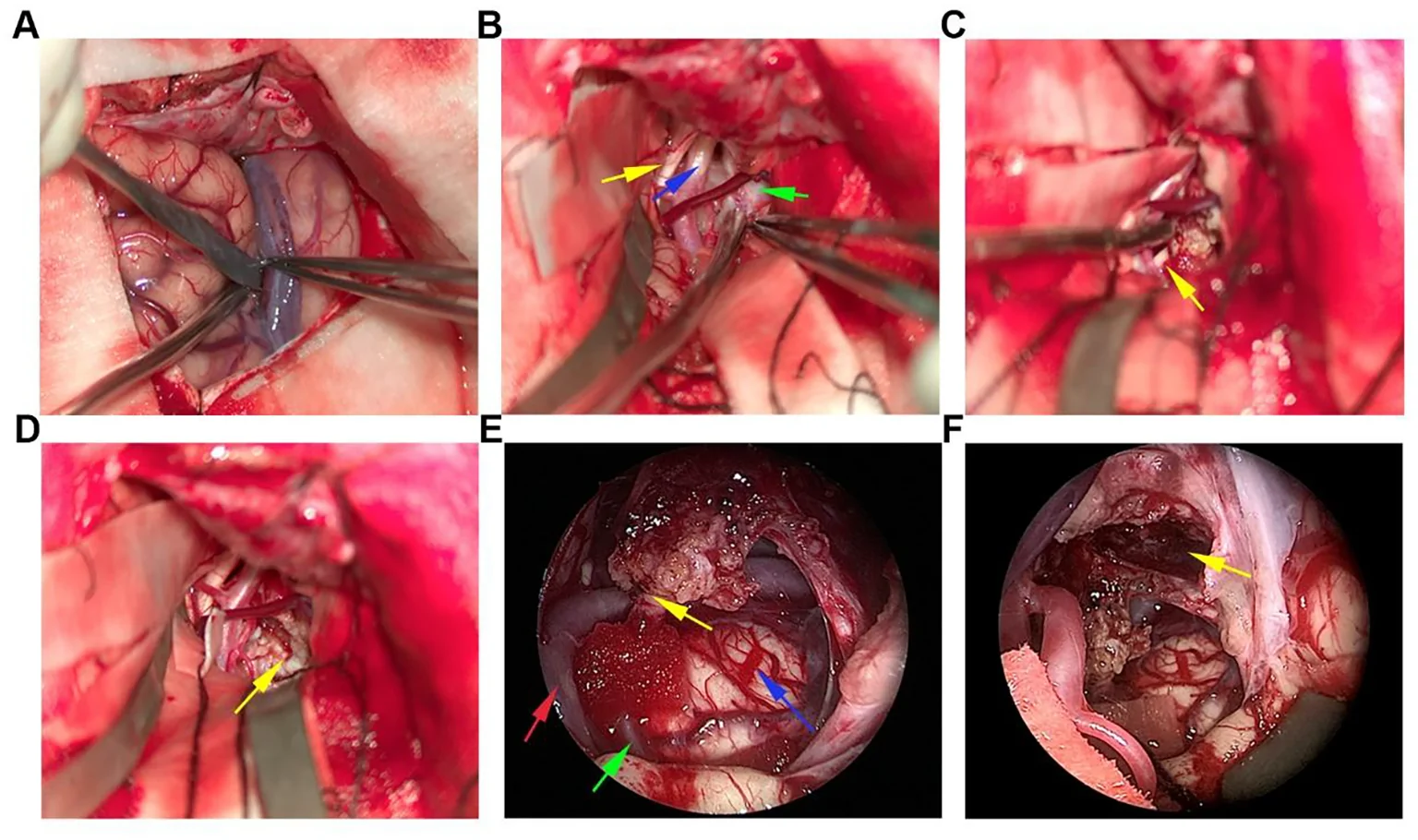
**(A)** Dissection of the Sylvian fissure to expose the surgical corridor. **(B)** Intraoperative exposure of the right optic nerve (yellow arrow), internal carotid artery (blue arrow), and lateral wall of the cavernous sinus (green arrow). **(C)** Visualization of the tumor originating from the oculomotor nerve; the arrow indicates the oculomotor nerve. **(D)** Piecemeal resection of the tumor tissue. **(E)** Neuroendoscopic exploration demonstrating the nerve–residual tumor complex (yellow arrow) coursing between the posterior cerebral artery (red arrow) and superior cerebellar artery (green arrow), consistent with the anatomical trajectory of the oculomotor nerve. Brainstem (blue arrow). **(F)** Neuroendoscopic inspection of the cavernous sinus cavity and curettage of residual tumor within the sinus (arrow indicates the cavernous sinus cavity).

Because the tumor had extended into the optic canal and lay adjacent to the brainstem, and circumferential encasement of the cavernous ICA with focal stenosis was already evident, the case was discussed in a multidisciplinary setting and microsurgical resection via a pterional approach was recommended over observation or primary radiosurgery. Following admission and completion of preoperative evaluations, the patient underwent microsurgical resection with endoscope assistance of the skull base lesion via a right pterional craniotomy under general anesthesia. Intraoperatively, the Sylvian fissure was dissected first (**Figure 2A**). The right frontal and temporal lobes were gently retracted, allowing sequential exposure and protection of the right optic nerve, olfactory nerve, internal carotid artery, A1 segment of the anterior cerebral artery, middle cerebral artery, and anterior choroidal artery (**Figure 2B**). Deep to the right internal carotid artery and the M1 segment of the middle cerebral artery, the tumor was visualized as a grayish-white, firm, moderately vascularized mass. The lesion exhibited intimate involvement and dense adhesion to the cisternal segment of the oculomotor nerve as it entered the cavernous sinus (**Figure 2C**).

The tumor capsule was incised at an area devoid of neurovascular structures. Initial intracapsular debulking was performed to achieve decompression (**Figure 2D**), followed by meticulous dissection along the tumor capsule. Surgical exploration confirmed tumor extension into the cavernous sinus through both its roof and posterior wall. At the site where the oculomotor nerve entered the sinus, the nerve itself appeared replaced by tumor, obscuring standard anatomical landmarks. Neuroendoscopic inspection demonstrated that the tumor-nerve complex was situated between the posterior cerebral artery (PCA, superiorly) and the superior cerebellar artery (SCA, inferiorly), with the brainstem posteriorly — a classic anatomical landmark for identifying the cisternal segment of cranial nerve III (**Figure 2E**). The tumor was inseparable from the substance of the nerve, and no distinct proximal or distal segments of CN III could be identified beyond the tumor borders, suggesting an intrinsic origin from CN III. We gained entry into the cavernous sinus by opening its roof and lateral wall along the posterior oculomotor trigone, then proceeded to excise the intracavernous portion of the lesion.

To address regions within the deep cavernous sinus that were beyond the limited visual field of the operating microscope, a 0-degree rigid neuroendoscope (Karl Storz, Germany) was introduced as an adjunct, held by an assistant while the surgeon performed bimanual microsurgical dissection. Under direct endoscopic visualization, residual tumor within the cavernous sinus was carefully removed by microsurgical dissection along an identifiable tumor–vessel interface (**Figure 2F**). Throughout this step, the internal carotid artery was protected by sharp dissection along a clear plane and the use of cottonoid pledgets for coverage, avoiding blind instrumentation in the vicinity of the vessel. The oculomotor nerve was preserved by gentle manipulation confined to the tumor capsule, with meticulous care taken to avoid traction or thermal injury. Intraoperative neurophysiological monitoring was not used in this case due to technical limitations. Given that the medial aspect of the tumor was intimately adherent to the cavernous segment of the internal carotid artery and the distal portion of the oculomotor nerve, gross total resection was not attempted in order to avoid neurovascular injury. As planned preoperatively, an intentional subtotal resection was performed, leaving a small residual component along the cavernous ICA to preserve oculomotor function.

### Postoperative course

**Figure 3:**
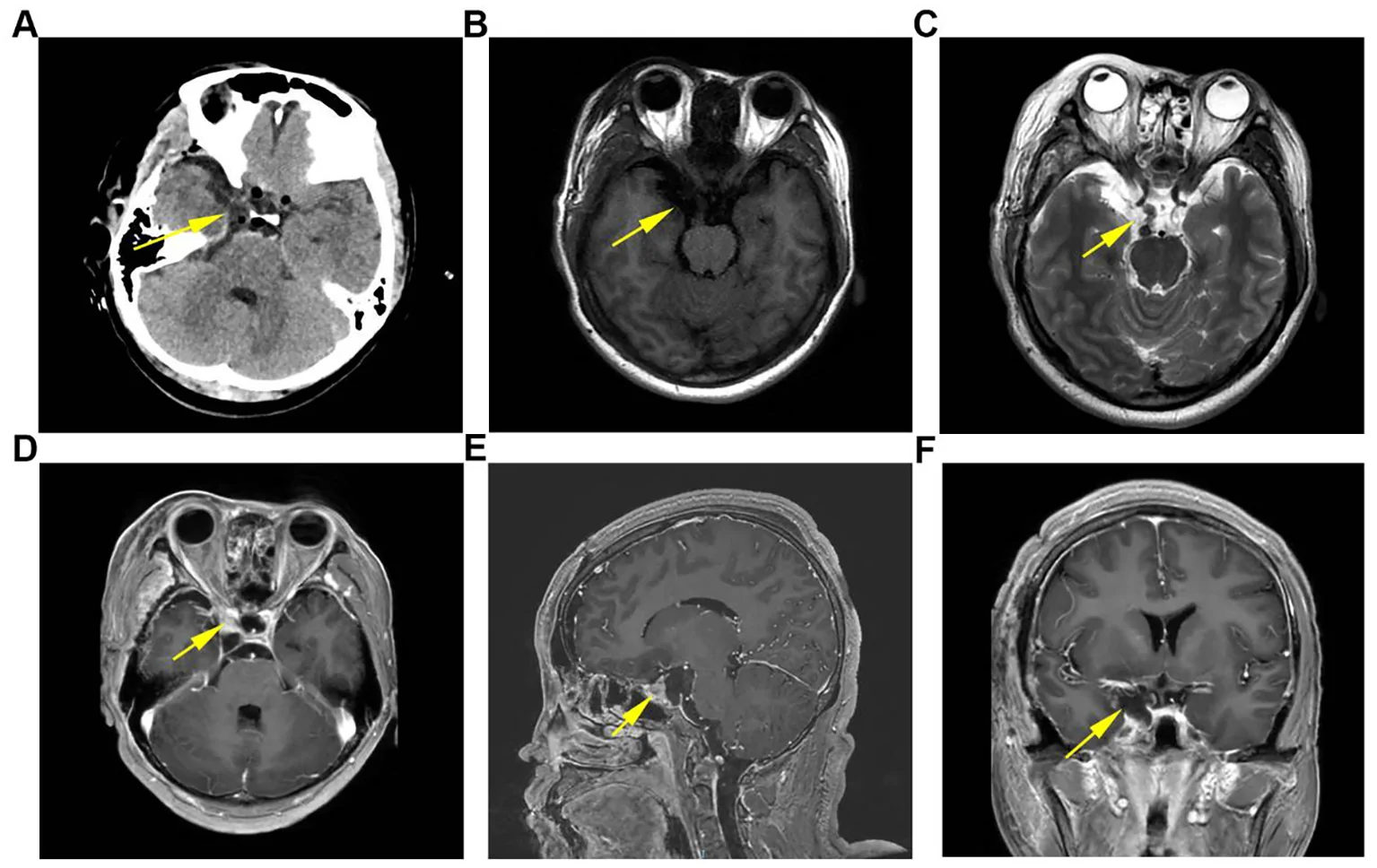
Postoperative imaging. **(A)** Axial non-contrast CT (brain window) showing no abnormal density at the surgical site. **(B)** Axial T1WI and **(C)** axial T2WI demonstrating no definite residual mass. **(D)** Axial and **(E)** sagittal CE-T1WI revealing a small residual enhancing focus in the right cavernous sinus (arrows). **(F)** Coronal CE-T1WI confirming near-total resection.

**Figure 4:**
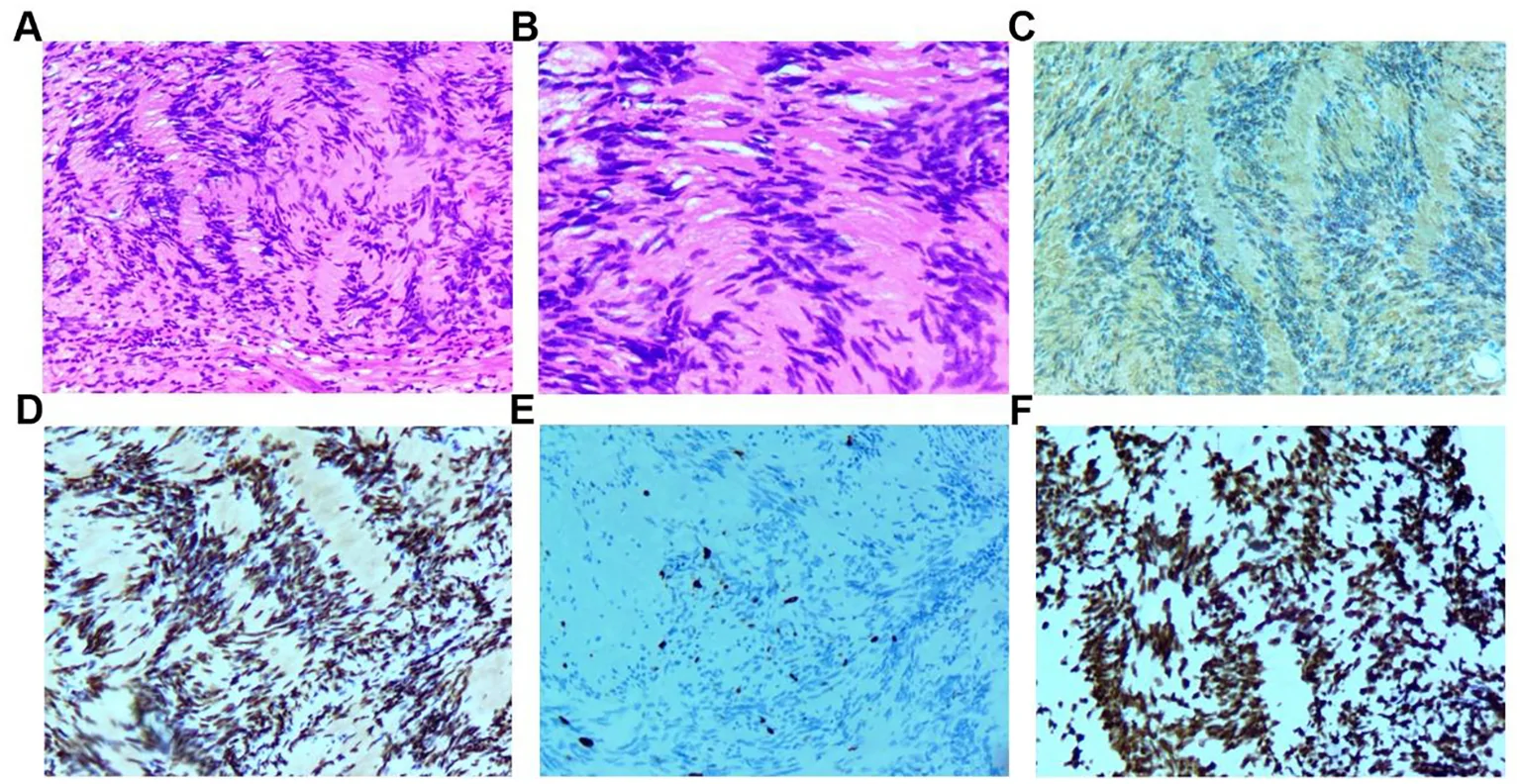
Histopathological and immunohistochemical features of the tumor. **(A)** Hematoxylin and eosin (H&E) staining (×100): Low-power view reveals a thin fibrous capsule and alternating areas of compact cellularity (Antoni A) and loose hypocellularity (Antoni B). **(B)** H&E staining (×200): High-power view demonstrates palisading arrangements of spindle-shaped tumor cells, forming Verocay bodies. **(C)** S-100 immunohistochemistry (×100): Diffuse and strong positive expression. **(D)** SOX10 immunohistochemistry (×100): Nuclear positive expression. **(E)** Ki-67 immunohistochemistry (×100): Proliferation index approximately 10%. **(F)** H3K27Me3 immunohistochemistry (×100): Retained expression (positive).

The patient’s postoperative recovery was uneventful. Right-sided ptosis showed partial improvement, with the vertical palpebral fissure height increasing from 5 mm preoperatively to 6 mm postoperatively. Complete resolution of ptosis was not achieved during the available follow-up period. Bilateral visual acuity also showed progressive improvement. Both pupils remained equal, round, and reactive to light without postoperative deterioration; full extraocular movements were preserved. Postoperative non-contrast CT obtained on postoperative day 1 and contrast-enhanced MRI performed on postoperative day 3 demonstrated an intentional subtotal resection, with a small residual enhancing focus identified in the right cavernous sinus (**Figures 3D, E**, arrows). This residual lesion represents the planned target for adjuvant stereotactic radiosurgery. Postoperative pathological examination revealed a spindle cell neoplasm, consistent with schwannoma based on immunohistochemical findings. Immunohistochemistry results were as follows: Vimentin (+), S-100 (+), SOX10 (+), EMA (-), PR (-), SSTR-2 (-), CD34 (focal +), STAT6 (-), H3K27Me3 (+), and Ki-67 (index 10%) (**Figure 4**). The Ki-67 proliferation index of approximately 10% represents an average index, the specimen was reviewed by a neuropathologist. Adjuvant SRS has been recommended for the residual tumor based on the documented residual enhancing focus, its cavernous sinus location, and multidisciplinary assessment, and the patient remains under follow-up.

## Discussion

An exceptionally uncommon benign neoplasm of the oculomotor nerve, oculomotor nerve schwannoma (ONS) arises from the Schwann cells investing this cranial nerve. Aberrant Schwann cell proliferation gives rise to schwannomas, which are generally benign in nature. However, malignant transformation can occur in rare instances, resulting in malignant peripheral nerve sheath tumor (MPNST) (10, 11). The clinical presentation of ONS is predominantly characterized by oculomotor nerve palsy, manifesting as ptosis, ocular motility deficits, and mydriasis. Some patients may also experience headache and visual deterioration (5, 6). The diagnosis of ONS poses a considerable challenge, and the condition is frequently misdiagnosed as other entities, such as recurrent painful ophthalmoplegic neuropathy (RPON) (12). Therefore, in patients with unexplained oculomotor nerve palsy, comprehensive neuroimaging should be performed to exclude an underlying ONS.

The imaging diagnosis of ONS primarily relies on MRI, particularly contrast-enhanced sequences. On MRI, these tumors typically appear as nodular or fusiform lesions following the trajectory of the third cranial nerve. They display iso- to hypointensity on T1WI, hyperintensity on T2WI, and avid post-contrast enhancement (6). In the present case, MRI revealed a space-occupying lesion in the right parasellar–cavernous sinus region, with prominent homogeneous enhancement of the solid component and scattered small non-enhancing cystic areas—features consistent with schwannoma. Additionally, proton magnetic resonance spectroscopy (¹H-MRS) may aid in differentiating benign from malignant tumors, with malignant lesions typically demonstrating elevated choline peaks and absent creatine and N-acetylaspartate peaks (10). Diffusion tensor imaging (DTI) can delineate the relationship between the tumor and adjacent nerve fibers, thereby providing valuable information for preoperative planning (13). Computed tomography (CT) also plays a role, albeit with lower sensitivity than MRI. On CT, schwannomas usually appear isodense or slightly hypodense, and may exhibit heterogeneous density due to intratumoral cystic changes or necrosis (14).

Histologically, ONS is classically characterized by alternating Antoni A areas (compact, spindle-shaped cells with Verocay body formation) and Antoni B areas (loosely arranged cells with myxoid degeneration) (15). Immunohistochemically, S-100 protein is a sensitive but not entirely specific marker for Schwann cell differentiation and is typically diffusely positive in benign ONS (16, 17). In contrast, MPNST may exhibit only partial or absent S-100 expression, accompanied by elevated expression of proliferative markers such as p53 and Ki-67 (18).

ONS must be differentiated from various other pathologies involving the oculomotor nerve. First, cavernous sinus meningiomas typically demonstrate a broad dural attachment and may exhibit a “dural tail” sign on contrast-enhanced imaging, with immunohistochemical positivity for epithelial membrane antigen (EMA); in contrast, ONS lacks dural attachment and is S-100 positive (19, 20). Second, posterior communicating artery aneurysms can compress the oculomotor nerve and cause palsy; CT angiography (CTA) or MR angiography (MRA) can reveal the vascular nature of the lesion, distinguishing it from the solid tumor appearance of ONS (21). Third, Tolosa-Hunt syndrome typically presents with acute painful ophthalmoplegia and shows a favorable response to corticosteroid therapy, whereas recurrent painful ophthalmoplegic neuropathy (RPON) is characterized by repeated episodes and a less consistent steroid response. ONS, by contrast, follows an indolent, progressive course and does not respond to steroids (22). Furthermore, schwannomas associated with neurofibromatosis frequently involve multiple cranial nerves (e.g., bilateral vestibular schwannomas), and genetic testing (NF2 mutation) can confirm the diagnosis (23).

For ONS, the mainstay of management is surgical excision, which aims to maximize tumor removal while safeguarding neurological integrity. The optimal approach is dictated by the anatomical location of the lesion. Cavernous sinus tumors are typically accessed via a pterional craniotomy or an endoscopic endonasal corridor; those confined to the orbit may be addressed through a lateral orbitotomy; and dumbbell-shaped lesions with both intracranial and extracranial extension frequently warrant combined skull base approaches (24, 25). Intraoperative neurophysiological monitoring (e.g., electromyography, somatosensory evoked potentials) enables real-time assessment of nerve function and reduces the risk of iatrogenic injury (26). In general, the crux of surgical management lies in striking a balance between the extent of resection and functional preservation. For large or complex ONS, a strategy of subtotal resection followed by adjuvant radiotherapy is a reasonable option (27). In the present case, a right pterional craniotomy was performed, and the cavernous sinus roof was opened via the posterior portion of the oculomotor trigone—a safe anatomical corridor into the cavernous sinus. Endoscope-assisted microsurgery played a key role in this procedure. The operating microscope provides excellent stereoscopic visualization and fine dexterity, making it ideal for resection of the main tumor bulk and identification of critical neurovascular structures. However, for deep regions within the cavernous sinus and areas lateral to the internal carotid artery that are beyond the direct line of sight of the microscope, conventional microsurgery is hampered by blind spots. The neuroendoscope offers the advantages of close-up inspection, a wide-angle field of view, and superior illumination. In this patient, it facilitated inspection of areas not directly visible through the microscope and assisted in the removal of residual tumor under direct visual control. In the present case, an intentional subtotal resection was preoperatively planned and intraoperatively executed for function preservation. Given the intimate adherence of the tumor to the cavernous segment of the internal carotid artery and the distal oculomotor nerve, complete resection was intentionally foregone to avoid permanent neurological morbidity. This decision is consistent with the current approach for skull base schwannomas, which prioritizes maximal safe resection with cranial nerve preservation over radicality at any cost, as endorsed by the EANS skull base consensus on non-vestibular schwannomas (28). In this case, after microsurgical debulking, neuroendoscopy was introduced to inspect the deep cavernous sinus, enabling curettage of residual intracavernous tumor while clearly delineating the relationship between the distal oculomotor nerve and the internal carotid artery, thereby avoiding blind maneuvers that could have resulted in neurovascular injury.

Stereotactic radiosurgery has become an established adjuvant treatment for skull base schwannomas, particularly for residual tumor in high-risk locations such as the cavernous sinus. According to a multicenter series, SRS achieves crude tumor control in 92% of ONS cases, with actuarial rates of 96% at 1 year and 86% at 10 years, supporting its role as an adjunctive treatment option for selected patients (29). In the present case, the Ki-67 proliferation index was approximately 10% as an average index across representative tumor areas, which is somewhat higher than the typical value for conventional schwannomas (usually <5%). However, this finding was interpreted together with the morphological and immunohistochemical features: the tumor showed no necrosis, no hypercellularity, no nuclear atypia, and no infiltrative growth, with retained H3K27me3 and diffuse S-100/SOX10 expression, supporting a benign schwannoma rather than cellular schwannoma or malignant peripheral nerve sheath tumor. Accordingly, the recommendation for adjuvant SRS was not based primarily on the Ki-67 index, but rather on the documented residual enhancing focus within the cavernous sinus, its high-risk location adjacent to the ICA and distal CN III, and the consensus of the multidisciplinary team. This approach is consistent with the strategy of maximal safe cytoreduction followed by radiosurgery for planned residuals in high-risk locations such as the cavernous sinus. In pediatric patients, fractionated stereotactic radiotherapy (SRT) may reduce the risk of radiation-induced injury; for example, two children aged 8 and 10 years were treated with 45–50 Gy in 25 fractions, and at 57-month follow-up, no tumor progression or neurocognitive deficits were observed (30).

The main limitation is the short follow-up to date; the long-term response of the residual tumor to the recommended adjuvant SRS remains to be observed. As a single case report, extrapolation of the findings should be undertaken with caution. Nevertheless, this case illustrates the application of endoscope-assisted microsurgery in the surgical management of complex ONS involving the cavernous sinus and highlights the importance of functional preservation through a planned near-total resection strategy, combined with adjuvant radiosurgery for the planned residual. Further follow-up is required to assess the long-term functional and oncological outcomes of this approach.

## Conclusion

We report an exceedingly rare case of primary oculomotor nerve schwannoma with cavernous sinus involvement. Via a right pterional craniotomy and employing a microsurgical resection with endoscope assistance, a planned function-preserving intentional subtotal resection was achieved through the oculomotor trigone of the cavernous sinus, with meticulous preservation of the oculomotor nerve and the cavernous segment of the internal carotid artery. Postoperatively, the patient exhibited notable improvement in ptosis and visual impairment, with partial recovery of oculomotor nerve function. This case demonstrates that the right pterional approach augmented by the endoscopic assistance effectively addresses the blind spots inherent to deep cavernous sinus surgery, thereby enabling maximal safe resection while reducing the risk of neurological morbidity. For the residual enhancing focus that could not be safely resected, adjuvant SRS has been recommended as a potential supplementary treatment. This strategy is consistent with current approaches combining maximal safe resection with radiosurgery for planned residuals in high-risk areas, although its long-term efficacy in this patient remains to be determined. Although follow-up remains short and adjuvant SRS has been recommended but not yet documented, the surgical strategy and the principle of prioritizing functional preservation illustrated in this case may inform the management of this rare cavernous sinus pathology.

## Data Availability

The original contributions presented in the study are included in the article/supplementary material. Further inquiries can be directed to the corresponding authors.
